# Fisher–Rao Distance for Finite-Energy Signal Manifolds: Geometric Foundations and Numerical Analysis

**DOI:** 10.3390/e28050569

**Published:** 2026-05-19

**Authors:** Franck Florin

**Affiliations:** Thales, 92190 Meudon, France; franck.florin@fr.thalesgroup.com

**Keywords:** Fisher–Rao distance, finite-energy signals, information geometry, statistical manifolds, Christoffel symbols, geodesic equations

## Abstract

This paper introduces a geometric framework for analyzing finite-energy signals observed with additive noise by representing them as points on statistical manifolds equipped with the Fisher–Rao metric. Each signal is associated with a parameter vector ***θ***, which defines a unique probability distribution p(x|θ) on a statistical manifold. We propose a unified approach based on the normal multivariate model to describe a raw signal mixed with additive stationary noise. In the approach considered, the background noise is typically assumed to be stationary, whereas the unknown signal is regarded as deterministic. Leveraging tools from information geometry, we compute geodesic equations for the statistical manifolds. We re-derive known results regarding the multivariate normal models and extend them to the signal processing domain. We show that in some cases, the geodesic equations can be solved to obtain a closed-form expression of the Fisher–Rao distance. This expression corresponds to a minimum bound when the sub-manifold is not geodesic, revealing a fundamental geometric constraint in signal parameter estimation. We introduce the spectral distance function, which characterizes the influence of each spectral component of the signals on the Fisher–Rao distance. Our findings provide theoretical insights for signal clustering and machine learning applications, where geometric distances can characterize classification and estimation tasks.

## 1. Introduction

Information geometry bridges differential geometry and statistical inference, enabling the rigorous analysis of parametric probability distributions as statistical manifolds [1]. Time series analysis and signal processing are well-established applications of information geometry. Modeling time series as realizations of statistical populations via parametric probability distributions enables the utilization of tools from differential geometry. Information geometry has been extensively applied to multivariate normal models [2,3,4,5,6,7,8]. With regard to signal analysis, stationary Gaussian time series are commonly examined via the statistical properties of their Fourier expansions. For instance, Amari and Nagaoka have derived the geometry of signal processing from the spectral density function, under the assumption that the sum of the squared logarithms of the power spectrum norm is finite [9] (p. 116). Extension to the modeling of finite-energy signals in additive noise remained underexplored until recently. A recent work addressed this gap by adapting the model to finite-energy signal manifolds. The representation of signals through their Fourier expansions can be used for finite-energy signals with a possible finite duration. The Fourier expansions are seen as random variables following multivariate normal distributions. Two Riemannian manifolds for finite-energy signals have been defined: the full manifold of all finite-energy signals in a given bandwidth and the sub-manifold of finite-energy signals with a known magnitude spectrum [10,11,12]. This previous work derived closed-form expressions of Fisher–Rao distances on these manifolds via resolution of the geodesic equations, analyzing asymptotic behaviors and demonstrating that the sub-manifold is not fully geodesic (i.e., the distances on the sub-manifold exceed those on the full manifold).

Here, we address again the case of finite-energy signal manifolds already discussed in [10,11,12] with dual objectives. First, we put into perspective the results already obtained with the methods of information geometry by recalling some classical formulas and providing new evidence. The objective of this first part is to explore the geometric foundations for the finite-energy signal manifolds. The elements are treated and detailed in Section 2, Section 3, Section 4 and Section 5. Although geodesic equations on the multivariate normal manifold have already been proven [4,5], we propose here a unified approach based on a common method of information geometry used by the research community [1,6,7,8,13]. In order to make the article self-contained, we develop new proofs for the geodesic equations and Fisher–Rao distance expressions on the multivariate normal manifold, aside from references to former works.

The second objective, addressed in the Section 6 and Section 7, is to analyze the numerical behavior of the Fisher–Rao distance between finite-energy signal observations. This section provides new examples not examined in the previous publications and highlights the new concept of spectral distance. We analyze the dependence of Fisher–Rao distance values with the signal-to-noise ratio, signal bandwidth and other factors as phase variations. To demonstrate that the proposed distance gives a practical advantage for signal estimation, we use a small toy problem.

Section 2 presents the method of information geometry commonly used to compute the Fisher–Rao distance based on the Fisher metric. This section references previous work by the research community [1,6,7,13]. We recall the general approach for deriving the geodesic equations. This section also provides an opportunity to introduce relevant definitions and notation conventions.

Section 3 reviews key contributions from previous research on the Riemannian geometry of the multivariate normal model, with a particular focus on the computation of geodesic distances [2,3,4,5,6]. Based on the method explained in Section 2, new proofs of the already known theorems are redeveloped in order to make the development self-contained.

Section 4 presents the proposed signal model and the associated statistical distributions. This signal model was previously introduced in earlier work on finite-energy signals [10,11,12,14]. The model is completed here to cover several signal cases. The parametric modeling is discussed in detail, leading to the definition of the manifolds L2(B,θ).

Section 5 addresses the general situation of a parametric model describing a family of finite-energy signals in any manifold L2(B,θ). We outline the various steps required to compute the Fisher–Rao distance, including the formulation of the differential equations for geodesics. General formulas for the Fisher–Rao distance on the manifolds identified in Section 4 are provided. The calculations of the Christoffel symbols are detailed.

Section 6 focuses on a specific manifold L2(B), which includes all other manifolds L2(B,θ) as sub-manifolds. The geodesic equations and a closed-form expression of the Fisher–Rao distance are derived for the manifold L2(B). This closed-form expression allows defining the spectral distance function $f_{\theta_{1},\theta_{2}}\left(\nu \right)$ and highlighting its relation to the Fisher–Rao distance.

Section 7 evaluates the Fisher–Rao distance on the previously defined manifold L2(B). The Fisher–Rao distance on this manifold is a minimum bound for the Fisher–Rao distances on all other manifolds L2(B,θ). We analyze the distance on the manifold and examine the asymptotic behaviors. We use numerical applications to compute the distance. Figures are drawn to illustrate the numerical behavior with some examples of signals.

The conclusion summarizes the main findings, discusses the relevance and limitations of the work, and outlines directions for future research.

## 2. How to Compute the Fisher–Rao Distance

### 2.1. Notation Conventions

Before describing the methodology for computing the Fisher–Rao distance, we introduce the notation conventions that will be employed throughout this paper.

Vectors and matrices are denoted in bold, while scalar values are presented in regular typeface. For example, ${\left[0\right]}_{P}$ represents the zero vector with *P* components. The superscript T denotes the transpose operation as in the expression $a^{T}$, where ***a*** is a matrix or a vector.

We note that $e_{i}$ the canonical unit vector where all the components are zero, except the ith component, which is equal to one:

$$\left\{\begin{array}{c} e_{i}^{j}=0,\;i\neq j\\ e_{i}^{j}=1,\;i=j \end{array}\right.$$

We note that $E_{\mathrm{ij}}$ is the symmetric canonical matrix where all the components are zero, except for the ijth and jith components, which are equal to one:

$$\left\{\begin{array}{c} E_{\mathrm{ij}}=e_{i}{e_{j}}^{T}+e_{j}{e_{i}}^{T},\;i\neq j\\ E_{\mathrm{ii}}=e_{i}{e_{i}}^{T},\;i=j \end{array}\right.$$

Here, $\delta_{i}^{j}$ is the Kronecker symbol (=1 when i=j and =0 otherwise).

$Re\left\{c\right\}$ and $Im\left\{c\right\}$ design the real and imaginary parts of the complex number *c*, respectively, where $c=Re\left\{c\right\}+ı\cdot Im\left\{c\right\}$ with ${\left(ı\right)}^{2}=-1$. The superscript * denotes the conjugate operation as in the expression $c^{*}=Re\left\{c\right\}-ı\cdot Im\left\{c\right\}$.

$Tr\left(A\right)$, $\det\left(A\right)$ and $A^{-1}$ design the trace, the determinant and the inverse matrix (when it exists) of matrix A, respectively.

We adopted Einstein’s summation convention to omit summation signs in tensor expressions, which allowed a concise representation of tensor operations. However, when summing over indices, such as the frequency, where the indexation is not strictly tensorial, we retained the explicit summation symbol, as shown in Equation (36).

With regard to the parameters, it is important to distinguish between $\theta_{2}$, which refers to a particular value of the parameter vector θ, and $\theta^{2}$, which denotes the second component of vector θ.

To avoid any ambiguity between exponentiation and the superscript index 2, exponents are written in explicit parentheses; for example, ${\left(\theta^{2}\right)}^{2}$ denotes the square of the component $\theta^{2}$.

Where appropriate, the vector $\theta =\left(\begin{array}{c} \phi \\ \varphi \end{array}\right)$ can be decomposed into its sub-components ϕ and φ, each with their respective indices:

- θ is a real vector of dimension *N*;
- ϕ is a real vector with a length *P* indexed by *u*, *v* or *w*, where 1≤u,v,w≤P;
- φ is a real vector with (N−P) components indexed by *q*, *r* or *p*, where P+1≤q,r,p≤N.

This convention facilitates a Schouten-like notation for differentiation: $\partial_{u}=\frac{\partial }{\partial \phi^{u}}$, $\partial_{v}=\frac{\partial }{\partial \phi^{v}}$, $\partial_{w}=\frac{\partial }{\partial \phi^{w}}$, $\partial_{q}=\frac{\partial }{\partial \varphi^{q}}$, $\partial_{r}=\frac{\partial }{\partial \varphi^{r}}$ and $\partial_{p}=\frac{\partial }{\partial \varphi^{p}}$.

The gradient with respect to the parameters is, by definition, the operator:

$${▽}_{\theta }={\left[\frac{\partial }{\partial \theta^{i}}\right]}_{i\in \{1,2\ldots N\}}$$

Under the definition of θ, ϕ and φ, we have

$$\begin{array}{cc} \; & {▽}_{\phi }={\left[\frac{\partial }{\partial \phi^{u}}\right]}_{u\in \{1,2\ldots P\}}\\ \; & {▽}_{\varphi }={\left[\frac{\partial }{\partial \varphi^{q}}\right]}_{q\in \{P+1,P+2\ldots N\}}\\ \; & {▽}_{\theta }=\left(\begin{array}{c} {▽}_{\phi }\\ {▽}_{\varphi } \end{array}\right) \end{array}$$

A specific case is the one where θ represents the parameters of a multivariate normal distribution with a mean μ and covariance matrix **Σ**. Assuming that μ is of a dimension *n*, the covariance matrix **Σ** is definite positive and can be represented by n(n+1)/2 coefficients. In this case, we can assume that N=n+n(n+1)/2 and P=n. For this situation, each parameter index *q* corresponds to a couple of indices (i,j) with 0≤i≤j≤n defining a component of the matrix **Σ**. With this notation, we can note that $E_{q}=E_{\mathrm{ij}}$.

### 2.2. Methodology to Compute the Fisher–Rao Distance

With the Fisher metric and the Levi-Civita connection, the geodesic is the curve of minimal length connecting two points. This minimal length defines a metric distance, known as the Fisher–Rao distance [3]. Using the Fisher metric, one may evaluate this statistical distance between two points, characterized by their parameter vectors $\theta_{1}$ and $\theta_{2}$. This distance quantifies the dissimilarity between the two statistical populations described by the probability distributions $p(x|\theta_{1})$ and $p(x|\theta_{2})$ [1]. The Fisher–Rao distance can be used for statistical analysis, including data clustering [15] and deep learning, to name but a few [2].

For conciseness, albeit at the risk of slight imprecision, the Fisher–Rao distance between the statistical distributions corresponding to two signals parameterized by $\theta_{1}$ and $\theta_{2}$ will hereafter simply be referred to as the distance between the two points $\theta_{1}$ and $\theta_{2}$. Our objective is to compute the Fisher–Rao distance between two points, $\theta_{1}$ and $\theta_{2}$, on the statistical manifold.

The Fisher–Rao distance computation follows the methodology described in several foundational references [1,9,13] and is widely used in the information geometry community [6,7,10,11,12]. The steps are as follows.

The first step is to specify a parametric model that describes the distribution of the observations. Let ***x*** denote the observation vector and ***θ*** denote the parameter vector. The stochastic behavior of the observations is characterized by the conditional probability distribution p(x|θ). As detailed in [9,16], the set of all such parametric probability distributions forms a statistical model, which can be regarded as a statistical manifold.

The Fisher information matrix $\left[g_{\mathrm{ij}}\right]$ is derived from the log-likelihood as follows:

(1) $$g_{ij}=E_{\theta }\left[\frac{\partial }{\partial \theta^{i}}\ln p\left(x|\theta \right)\frac{\partial }{\partial \theta^{j}}\ln p\left(x|\theta \right)\right]$$

Based on the Fisher information matrix, the Christoffel symbols are then computed according to

(2) $$\forall \;m,i,j=1,\ldots ,N\;\;\;\;\Gamma_{ij,m}:=g_{mk}\Gamma_{ij}^{k}=\frac{1}{2}\left(\frac{\partial g_{jm}}{\partial \theta^{i}}+\frac{\partial g_{mi}}{\partial \theta^{j}}-\frac{\partial g_{ij}}{\partial \theta^{m}}\right)$$

Once the Christoffel symbols are known, they can be employed in the following differential equations, whose solutions correspond to the geodesics $\tilde{\theta }\left(\varsigma \right)$ joining $\theta_{1}=\tilde{\theta }\left(0\right)$ and $\theta_{2}=\tilde{\theta }\left(1\right)$ (with $\varsigma \in \left[0,1\right]$):

(3) $$\forall \;m=1,\ldots ,N\;\;\;g_{mk}\frac{d^{2}\theta^{k}}{d\varsigma^{2}}+\Gamma_{ij,m}\frac{d\theta^{i}}{d\varsigma }\frac{d\theta^{j}}{d\varsigma }=0$$

Given the expressions of geodesics $\tilde{\theta }\left(\varsigma \right)$, the Fisher–Rao distance between the distribution at $\theta_{1}=\tilde{\theta }\left(0\right)$ and that at $\theta_{2}=\tilde{\theta }\left(1\right)$ is given by the following integral:

(4) $$d\left(\theta_{1},\theta_{2}\right)=\int_{0}^{1}\sqrt{g_{ij}\frac{d{\tilde{\theta }}^{i}}{d\varsigma }\frac{d{\tilde{\theta }}^{j}}{d\varsigma }}d\varsigma$$

Constraints in the parametric model of probability distributions restrict the distribution to a sub-manifold, and the Fisher–Rao geodesic distance on the sub-manifold is generally larger than the Fisher–Rao geodesic distance measured on the global manifold.

## 3. The Case of Multivariate Normal Distributions

### 3.1. The General Case

Finding closed-form expressions for the Fisher–Rao distance is recognized as a non-trivial endeavor, and such expressions are only available for a limited number of families of probability distributions [7]. Here, we consider multivariate normal distributions N(μ,Σ) given by

(5) $$p\left(X|\mu ,\Sigma \right)=\frac{1}{2^{\frac{n}{2}}\pi^{\frac{n}{2}}\sqrt{\det\left(\Sigma \right)}}\exp\left(-\frac{{\left(X-\mu \right)}^{T}\Sigma^{-1}\left(X-\mu \right)}{2}\right)$$

Here, the following definitions apply:

- *n* is an integer, the number of components;
- ***X*** is a real random vector with *n* components;
- **Σ** is an n×n positive-definite matrix;
- ***μ*** is a real vector with *n* components.

When the parameters are all the coefficients of ***μ*** and **Σ**, geodesic equations corresponding to multivariate normal distributions N(μ,Σ) take the form of second-order ordinary differential equations (with ς∈[0,1]) [4,5,6]:

(6) $$\left\{\begin{array}{c} \frac{d^{2}\mu }{d\varsigma^{2}}-\frac{d\Sigma }{d\varsigma }\Sigma^{-1}\frac{d\mu }{d\varsigma }={\left[0\right]}_{n}\\ \frac{d^{2}\Sigma }{d\varsigma^{2}}+\frac{d\mu }{d\varsigma }\frac{d\mu^{T}}{d\varsigma }-\frac{d\Sigma }{d\varsigma }\Sigma^{-1}\frac{d\Sigma }{d\varsigma }={\left[0\right]}_{n\times n} \end{array}\right.$$

**Proof.**  The proof relies on the method explained in Section 2.

First, we compute the Fisher matrix. From Equation (5), we can write

$$\ln p\left(x|\theta \right)=-\frac{n}{2}\ln2\pi -\frac{1}{2}\ln\det(\Sigma )-\frac{1}{2}{\left(X-\mu \right)}^{T}\Sigma^{-1}\left(X-\mu \right)$$

Assuming that *u*, *v* and *w* represent the parameter indices (varying from 1 to P) of the mean ***μ*** and *q*, *r* and *p* represent the parameter indices (varying from P + 1 to N) of the covariance matrix **Σ**, we can write the following:

(7) $$\begin{array}{cc} \partial_{u}\ln p\left(x|\theta \right) & =\partial_{u}\mu^{T}\Sigma^{-1}\left(X-\mu \right)\;\;\;\;\;\;\;\;\;\;\; \end{array}$$

(8) $$\begin{array}{cc} \partial_{q}\ln p\left(x|\theta \right) & =-\frac{1}{2}\partial_{q}\ln\det(\Sigma )-\frac{1}{2}{\left(X-\mu \right)}^{T}\partial_{q}\Sigma^{-1}\left(X-\mu \right) \end{array}$$

From Equation (7), we can see that

$$\begin{array}{cc} \partial_{q}\partial_{u}\ln p\left(x|\theta \right) & =\partial_{u}\mu^{T}\partial_{q}\Sigma^{-1}\left(X-\mu \right) \end{array}$$

We know from [9] (p. 28) that the Fisher metric can also be written as $g_{qu}=E_{\theta }\left[-\partial_{q}\partial_{u}\ln p\left(x|\theta \right)\right]$. Thus, we can easily obtain

$$\begin{array}{cc} g_{qu} & =-\partial_{u}\mu^{T}\partial_{q}\Sigma^{-1}E_{\theta }\left[X-\mu \right]\\ \; & =0 \end{array}$$

As $g_{uv}=E_{\theta }\left[-\partial_{v}\partial_{u}\ln p\left(x|\theta \right)\right]$, we also have

$$\begin{array}{cc} g_{uv} & =-\partial_{u}\partial_{v}\mu^{T}\Sigma^{-1}E_{\theta }\left[X-\mu \right]+\partial_{u}\mu^{T}\Sigma^{-1}\partial_{v}\mu \\ \; & =\partial_{u}\mu^{T}\Sigma^{-1}\partial_{v}\mu \\ \; & =e_{u}^{T}\Sigma^{-1}e_{v} \end{array}$$

Knowing from algebra rules that $d\ln\det(A)=Tr\left(A^{-1}dA\right)$, $dA^{-1}=-A^{-1}dAA^{-1}$ and $Tr\left(\mathrm{AB}\right)=Tr\left(\mathrm{BA}\right)$, we can deduce from Equation (8) the following equations:

$$\begin{array}{cc} \partial_{q}\ln p\left(x|\theta \right) & =-\frac{1}{2}Tr\left(\Sigma^{-1}\partial_{q}\Sigma \right)+\frac{1}{2}{\left(X-\mu \right)}^{T}\Sigma^{-1}\partial_{q}\Sigma \Sigma^{-1}\left(X-\mu \right)\\ \; & =-\frac{1}{2}Tr\left(\Sigma^{-1}\partial_{q}\Sigma -{\left(X-\mu \right)}^{T}\Sigma^{-1}\partial_{q}\Sigma \Sigma^{-1}\left(X-\mu \right)\right)\\ \; & =-\frac{1}{2}Tr\left(\Sigma^{-1}\partial_{q}\Sigma -\Sigma^{-1}\partial_{q}\Sigma \Sigma^{-1}\left(X-\mu \right){\left(X-\mu \right)}^{T}\right)\\ \; & =-\frac{1}{2}Tr\left(\partial_{q}\Sigma \Sigma^{-1}\left(\Sigma -\left(X-\mu \right){\left(X-\mu \right)}^{T}\right)\Sigma^{-1}\right) \end{array}$$

As each *q* corresponds to a couple of indices (i,j) with i≤j defining a component of the matrix **Σ**, we can note $E_{q}=E_{\mathrm{ij}}$, and we have the following:

(9) $$\partial_{q}\Sigma =E_{q}$$

and

(10) $$\partial_{q}\Sigma^{-1}=-\Sigma^{-1}E_{q}\Sigma^{-1}$$

This leads to the following equations:

$$\begin{array}{cc} \partial_{q}\ln p\left(x|\theta \right) & =-\frac{1}{2}Tr\left(E_{q}\Sigma^{-1}\left(\Sigma -\left(X-\mu \right){\left(X-\mu \right)}^{T}\right)\Sigma^{-1}\right)\\ \; & =-\frac{1}{2}Tr\left(E_{q}\Sigma^{-1}-E_{q}\Sigma^{-1}\left(X-\mu \right){\left(X-\mu \right)}^{T}\Sigma^{-1}\right) \end{array}$$

With *r* corresponding to a couple (k,l) of parameter indices in the matrix **Σ** with k≤l, we can write

$$\begin{array}{cc} \partial_{r}\partial_{q}\ln p\left(x|\theta \right)= & -\frac{1}{2}Tr\left(E_{q}\partial_{r}\Sigma^{-1}\right)\\ & +\frac{1}{2}Tr\left(E_{q}\partial_{r}\Sigma^{-1}\left(X-\mu \right){\left(X-\mu \right)}^{T}\Sigma^{-1}\right)\\ \; & +\frac{1}{2}Tr\left(E_{q}\Sigma^{-1}\left(X-\mu \right){\left(X-\mu \right)}^{T}\partial_{r}\Sigma^{-1}\right) \end{array}$$

We know from [9] (p. 28) that the Fisher metric can also be writen as $g_{rq}=E_{\theta }\left[-\partial_{r}\partial_{q}\ln p\left(x|\theta \right)\right]$, and by definition, the covariance matrix $\Sigma =E_{\theta }\left[\left(X-\mu \right){\left(X-\mu \right)}^{T}\right]$. Therefore, we can write the following:

$$\begin{array}{cc} g_{rq}= & +\frac{1}{2}Tr\left(E_{q}\partial_{r}\Sigma^{-1}\right)\\ & -\frac{1}{2}Tr\left(E_{q}\partial_{r}\Sigma^{-1}\Sigma \Sigma^{-1}\right)\\ \; & -\frac{1}{2}Tr\left(E_{q}\Sigma^{-1}\Sigma \partial_{r}\Sigma^{-1}\right)\\ = & -\frac{1}{2}Tr\left(E_{q}\partial_{r}\Sigma^{-1}\right)\\ = & -\frac{1}{2}Tr\left(E_{q}\left(-\Sigma^{-1}\partial_{r}\Sigma \Sigma^{-1}\right)\right)\\ = & \frac{1}{2}Tr\left(E_{q}\Sigma^{-1}E_{r}\Sigma^{-1}\right) \end{array}$$

As a consequence, the Fisher matrix is a block diagonal matrix:

(11) $$\left[g_{\mathrm{ij}}\right]=\left(\begin{array}{cc} \left[g_{\mathrm{uv}}\right] & \left[0\right]\\ \left[0\right] & \left[g_{\mathrm{qr}}\right] \end{array}\right)$$

with

(12) $$\begin{array}{cc} g_{uv} & =e_{u}^{T}\Sigma^{-1}e_{v}\;\;\;\; \end{array}$$

(13) $$\begin{array}{cc} g_{qu} & =0\;\;\;\;\;\; \end{array}$$

(14) $$\begin{array}{cc} \;\;g_{rq} & =\frac{1}{2}Tr\left(E_{q}\Sigma^{-1}E_{r}\Sigma^{-1}\right) \end{array}$$

The second step of the proof consists of computing the $\Gamma_{ij,m}$.

Regarding $\Gamma_{ij,m}$, from Equation (2), we have the following relationships:

$$\begin{array}{cc} \partial_{w}g_{uv} & =\partial_{w}\left(e_{u}^{T}\Sigma^{-1}e_{v}\right)\\ \; & =0\\ \partial_{w}g_{rq} & =\frac{1}{2}Tr\left(\partial_{w}\left(E_{q}\Sigma^{-1}E_{r}\Sigma^{-1}\right)\right)\\ \; & =0\\ \partial_{w}g_{qu} & =0 \end{array}$$

With *p* corresponding to a couple (f,g) of parameter indices in the matrix **Σ** with f≤g, we can write

$$\begin{array}{cc} \partial_{p}g_{uv} & =e_{u}^{T}\partial_{p}\Sigma^{-1}e_{v}\\ \; & =-e_{u}^{T}\Sigma^{-1}E_{p}\Sigma^{-1}e_{v}\\ \partial_{p}g_{qu} & =0\\ \partial_{p}g_{rq} & =\frac{1}{2}Tr\left(\partial_{p}\left(E_{q}\Sigma^{-1}E_{r}\Sigma^{-1}\right)\right)\\ \; & =\frac{1}{2}Tr\left(E_{q}\partial_{p}\Sigma^{-1}E_{r}\Sigma^{-1}+E_{q}\Sigma^{-1}E_{r}\partial_{p}\Sigma^{-1}\right)\\ \; & =-\frac{1}{2}Tr\left(E_{q}\Sigma^{-1}E_{p}\Sigma^{-1}E_{r}\Sigma^{-1}+E_{q}\Sigma^{-1}E_{r}\Sigma^{-1}E_{p}\Sigma^{-1}\right)\\ \; & =-\frac{1}{2}Tr\left(E_{q}\Sigma^{-1}E_{p}\Sigma^{-1}E_{r}\Sigma^{-1}+\Sigma^{-1}E_{p}\Sigma^{-1}E_{r}\Sigma^{-1}E_{q}\right)\\ \; & =-\frac{1}{2}Tr\left(E_{q}\Sigma^{-1}E_{p}\Sigma^{-1}E_{r}\Sigma^{-1}+E_{q}\Sigma^{-1}E_{p}\Sigma^{-1}E_{r}\Sigma^{-1}\right)\\ \; & =-Tr\left(E_{q}\Sigma^{-1}E_{p}\Sigma^{-1}E_{r}\Sigma^{-1}\right)\\ \; & =\partial_{q}g_{pr}=\partial_{r}g_{pq} \end{array}$$

As a consequence, Equation (2) can be expressed as follows:

$$\begin{array}{cc} \Gamma_{uv,w} & =0\\ \Gamma_{uv,q} & =\frac{1}{2}\left(\partial_{u}g_{vq}+\partial_{v}g_{uq}-\partial_{q}g_{uv}\right)\\ \; & =\frac{1}{2}\left(0+0+e_{u}^{T}\Sigma^{-1}E_{q}\Sigma^{-1}e_{v}\right)\\ \; & =\frac{1}{2}e_{u}^{T}\Sigma^{-1}E_{q}\Sigma^{-1}e_{v}\\ \Gamma_{uq,w} & =\frac{1}{2}\left(\partial_{u}g_{wq}+\partial_{q}g_{uw}-\partial_{w}g_{uq}\right)\\ \; & =\frac{1}{2}\left(0+\partial_{q}g_{uw}-0\right)\\ \; & =\frac{1}{2}\left(0-e_{u}^{T}\Sigma^{-1}E_{q}\Sigma^{-1}e_{w}-0\right)\\ \; & =-\frac{1}{2}e_{u}^{T}\Sigma^{-1}E_{q}\Sigma^{-1}e_{w} \end{array}$$

$$\begin{array}{cc} \Gamma_{qu,w} & =\frac{1}{2}\left(\partial_{q}g_{wu}+\partial_{u}g_{qw}-\partial_{w}g_{qu}\right)\\ \; & =\frac{1}{2}\left(\partial_{q}g_{wu}+0-0\right)\\ \; & =\frac{1}{2}\left(-e_{w}^{T}\Sigma^{-1}E_{q}\Sigma^{-1}e_{u}+0-0\right)\\ \; & =-\frac{1}{2}e_{w}^{T}\Sigma^{-1}E_{q}\Sigma^{-1}e_{u}\\ \; & =\Gamma_{uq,w} \end{array}$$

$$\begin{array}{cc} \Gamma_{uq,r} & =\frac{1}{2}\left(\partial_{u}g_{qr}+\partial_{q}g_{ur}-\partial_{r}g_{uq}\right)\\ \; & =\frac{1}{2}\left(0+0-0\right)\\ \; & =0\\ \Gamma_{qr,w} & =0\\ \Gamma_{qr,p} & =\frac{1}{2}\left(\partial_{q}g_{pr}+\partial_{r}g_{qp}-\partial_{p}g_{qr}\right)\\ \; & =\frac{1}{2}\left(\partial_{q}g_{pr}+\partial_{r}g_{qp}-\partial_{p}g_{qr}\right)\\ \; & =\frac{1}{2}\left(\partial_{q}g_{pr}\right)\\ \; & =-\frac{1}{2}Tr\left(E_{q}\Sigma^{-1}E_{p}\Sigma^{-1}E_{r}\Sigma^{-1}\right) \end{array}$$

The third step of the proof consists of replacing the values of $\Gamma_{ij,m}$ in the geodesic equation (Equation (3)).

As a consequence, Equation (3) becomes

$$\begin{array}{c} \left\{\begin{array}{c} \forall u\;\;\;\;g_{uv}\frac{d^{2}\theta^{v}}{d\varsigma^{2}}+\Gamma_{wq,u}\frac{d\theta^{w}}{d\varsigma }\frac{d\theta^{q}}{d\varsigma }+\Gamma_{qw,u}\frac{d\theta^{q}}{d\varsigma }\frac{d\theta^{w}}{d\varsigma }=0\\ \forall q\;\;\;\;g_{qr}\frac{d^{2}\theta^{r}}{d\varsigma^{2}}+\Gamma_{uv,q}\frac{d\theta^{u}}{d\varsigma }\frac{d\theta^{v}}{d\varsigma }+\Gamma_{pr,q}\frac{d\theta^{p}}{d\varsigma }\frac{d\theta^{r}}{d\varsigma }=0 \end{array}\right. \end{array}$$

These equations can be rewritten as follows:

$$\begin{array}{c} \left\{\begin{array}{c} \forall u\;\;\;\;e_{u}^{T}\Sigma^{-1}e_{v}\frac{d^{2}\theta^{v}}{d\varsigma^{2}}-\frac{1}{2}e_{u}^{T}\Sigma^{-1}E_{q}\Sigma^{-1}e_{w}\frac{d\theta^{w}}{d\varsigma }\frac{d\theta^{q}}{d\varsigma }-\frac{1}{2}e_{u}^{T}\Sigma^{-1}E_{q}\Sigma^{-1}e_{w}\frac{d\theta^{w}}{d\varsigma }\frac{d\theta^{q}}{d\varsigma }=0\\ \forall q\;\;\;\;Tr\left(E_{q}\Sigma^{-1}E_{r}\Sigma^{-1}\right)\frac{d^{2}\theta^{r}}{d\varsigma^{2}}+e_{u}^{T}\Sigma^{-1}E_{q}\Sigma^{-1}e_{v}\frac{d\theta^{u}}{d\varsigma }\frac{d\theta^{v}}{d\varsigma }-Tr\left(E_{q}\Sigma^{-1}E_{p}\Sigma^{-1}E_{r}\Sigma^{-1}\right)\frac{d\theta^{p}}{d\varsigma }\frac{d\theta^{r}}{d\varsigma }=0 \end{array}\right. \end{array}$$

In other words, we have

$$\begin{array}{c} \left\{\begin{array}{c} \forall u\;\;\;\;e_{u}^{T}\Sigma^{-1}\left(e_{v}\frac{d^{2}\theta^{v}}{d\varsigma^{2}}-2\cdot \frac{1}{2}\frac{d\theta^{q}}{d\varsigma }E_{q}\Sigma^{-1}e_{w}\frac{d\theta^{w}}{d\varsigma }\right)=0\\ \forall q\;\;\;\;Tr\left(\Sigma^{-1}E_{q}\Sigma^{-1}E_{r}\frac{d^{2}\theta^{r}}{d\varsigma^{2}}+\Sigma^{-1}E_{q}\Sigma^{-1}e_{v}e_{u}^{T}\frac{d\theta^{u}}{d\varsigma }\frac{d\theta^{v}}{d\varsigma }-\Sigma^{-1}E_{q}\Sigma^{-1}E_{p}\Sigma^{-1}E_{r}\frac{d\theta^{p}}{d\varsigma }\frac{d\theta^{r}}{d\varsigma }\right)=0 \end{array}\right. \end{array}$$

and finally

$$\begin{array}{c} \left\{\begin{array}{c} \forall u\;\;\;\;e_{u}^{T}\Sigma^{-1}\left(e_{v}\frac{d^{2}\theta^{v}}{d\varsigma^{2}}-\frac{d\theta^{q}}{d\varsigma }E_{q}\Sigma^{-1}e_{w}\frac{d\theta^{w}}{d\varsigma }\right)=0\\ \forall q\;\;\;\;Tr\left[\Sigma^{-1}E_{q}\Sigma^{-1}\left(E_{r}\frac{d^{2}\theta^{r}}{d\varsigma^{2}}+\frac{d\theta^{v}}{d\varsigma }e_{v}e_{u}^{T}\frac{d\theta^{u}}{d\varsigma }-E_{p}\frac{d\theta^{p}}{d\varsigma }\Sigma^{-1}E_{r}\frac{d\theta^{r}}{d\varsigma }\right)\right]=0 \end{array}\right. \end{array}$$

We know that $a^{T}b$ defines a scalar product between the two vectors a and b, while $Tr[A^{T}B]$ defines a scalar product between the two matrices A and B.

As $\left\{\forall u\;\;\Sigma^{-1}e_{u}\right\}$ is the basis of $R^{n}$, and $\left\{\forall q\;\;\Sigma^{-1}E_{q}\Sigma^{-1}\right\}$ is a basis of the vector space of all symmetric n×n matrices, the two equations become

$$\begin{array}{c} \left\{\begin{array}{c} e_{v}\frac{d^{2}\theta^{v}}{d\varsigma^{2}}-\frac{d\theta^{q}}{d\varsigma }E_{q}\Sigma^{-1}e_{w}\frac{d\theta^{w}}{d\varsigma }={\left[0\right]}_{n}\\ E_{r}\frac{d^{2}\theta^{r}}{d\varsigma^{2}}+\frac{d\theta^{v}}{d\varsigma }e_{v}e_{u}^{T}\frac{d\theta^{u}}{d\varsigma }-E_{p}\frac{d\theta^{p}}{d\varsigma }\Sigma^{-1}E_{r}\frac{d\theta^{r}}{d\varsigma }={\left[0\right]}_{n\times n} \end{array}\right. \end{array}$$

Due to the definitions of $e_{v}$ and $E_{r}$, we can write

$$\begin{array}{c} \mu =\theta^{u}e_{u}\\ \Sigma =\theta^{q}E_{q} \end{array}$$

$$\begin{array}{c} \frac{d\mu }{d\varsigma }=\frac{d\theta^{u}}{d\varsigma }e_{u}\\ \frac{d\Sigma }{d\varsigma }=\frac{d\theta^{q}}{d\varsigma }E_{q} \end{array}$$

$$\begin{array}{c} \frac{d^{2}\mu }{d\varsigma^{2}}=\frac{d^{2}\theta^{u}}{d\varsigma^{2}}e_{u}\\ \frac{d^{2}\Sigma }{d\varsigma^{2}}=\frac{d^{2}\theta^{q}}{d\varsigma^{2}}E_{q} \end{array}$$

and the two geodesic equations can be written as follows:

$$\begin{array}{c} \left\{\begin{array}{c} \frac{d^{2}\mu }{d\varsigma^{2}}-\frac{d\Sigma }{d\varsigma }\Sigma^{-1}\frac{d\mu }{d\varsigma }={\left[0\right]}_{n}\\ \frac{d^{2}\Sigma }{d\varsigma^{2}}+\frac{d\mu }{d\varsigma }\frac{d\mu^{T}}{d\varsigma }-\frac{d\Sigma }{d\varsigma }\Sigma^{-1}\frac{d\Sigma }{d\varsigma }={\left[0\right]}_{n\times n} \end{array}\right. \end{array}$$

□

These geodesic equations have been solved by fixing the initial value conditions in ***μ*** and $\frac{d\mu }{dt}$ [3,8]. However, they were not solved in the general case for boundary conditions, i.e., fixing ***μ*** at ς=0, ς=1 ($\mu \left(0\right)=\mu_{1}$ and $\mu \left(1\right)=\mu_{2}$).

Once we have the expressions of the solutions of the geodesic equations $\tilde{\mu }$ and $\tilde{\Sigma }$, we can compute the Fisher–Rao distance using the following formula:

(15) $$d\left(\theta_{1},\theta_{2}\right)=\int_{0}^{1}\sqrt{{\frac{d\tilde{\mu }}{d\varsigma }}^{T}{\tilde{\Sigma }}^{-1}\frac{d\tilde{\mu }}{d\varsigma }-\frac{1}{2}Tr\left(\frac{d\tilde{\Sigma }}{d\varsigma }\frac{d{\tilde{\Sigma }}^{-1}}{d\varsigma }\right)}d\varsigma$$

**Proof.**  From Equations (4) and (11), we can write

$$\begin{array}{cc} d(\theta_{1},\theta_{2}) & =\int_{0}^{1}\sqrt{g_{uv}\frac{d{\tilde{\theta }}^{u}}{d\varsigma }\frac{d{\tilde{\theta }}^{v}}{d\varsigma }+g_{qr}\frac{d{\tilde{\theta }}^{q}}{d\varsigma }\frac{d{\tilde{\theta }}^{r}}{d\varsigma }}d\varsigma \\ \; & =\int_{0}^{1}\sqrt{\frac{d{\tilde{\theta }}^{u}}{d\varsigma }e_{u}^{T}{\tilde{\Sigma }}^{-1}e_{v}\frac{d{\tilde{\theta }}^{v}}{d\varsigma }+\frac{1}{2}Tr\left(\frac{d{\tilde{\theta }}^{q}}{d\varsigma }E_{q}{\tilde{\Sigma }}^{-1}\frac{d{\tilde{\theta }}^{r}}{d\varsigma }E_{r}{\tilde{\Sigma }}^{-1}\right)}d\varsigma \\ \; & =\int_{0}^{1}\sqrt{{\frac{d\tilde{\mu }}{d\varsigma }}^{T}{\tilde{\Sigma }}^{-1}\frac{d\tilde{\mu }}{d\varsigma }+\frac{1}{2}Tr\left(\frac{d\tilde{\Sigma }}{d\varsigma }{\tilde{\Sigma }}^{-1}\frac{d\tilde{\Sigma }}{d\varsigma }{\tilde{\Sigma }}^{-1}\right)}d\varsigma \\ \; & =\int_{0}^{1}\sqrt{{\frac{d\tilde{\mu }}{d\varsigma }}^{T}{\tilde{\Sigma }}^{-1}\frac{d\tilde{\mu }}{d\varsigma }-\frac{1}{2}Tr\left(\frac{d\tilde{\Sigma }}{d\varsigma }\frac{d{\tilde{\Sigma }}^{-1}}{d\varsigma }\right)}d\varsigma \end{array}$$

□

### 3.2. The Case Where Σ Is Constant

As stated above, in the general case, there is no explicit closed-form expression for the Fisher–Rao distance between multivariate normal probability distributions. Nevertheless, a closed-form expression can be derived in particular instances, such as multivariate normal distributions that share a common covariance matrix. The solutions for the equations with the boundary conditions are known for the distribution N(μ,Σ) in the case where Σ is the constant [6]. Below, we examine this case, which is of interest for the signal model.

When we consider the distribution N(μ,Σ) with the Σ constant, and when we have θ=μ, the second line of Equation (6) does not exist, and the geodesic equation becomes

(16) $$\frac{d^{2}\mu }{d\varsigma^{2}}={\left[0\right]}_{n}$$

**Proof.**  From Equation (5), we can write

$$\ln p\left(x|\theta \right)=-\frac{n}{2}\ln2\pi -\frac{1}{2}\ln\det(\Sigma )-\frac{1}{2}{\left(X-\mu \right)}^{T}\Sigma^{-1}\left(X-\mu \right)$$

Then, using the Leibniz rule, we have

$$\frac{\partial }{\partial \theta^{i}}\ln p\left(x|\theta \right)=-\frac{1}{2}{\left(-\frac{\partial \mu }{\partial \theta^{i}}\right)}^{T}\Sigma^{-1}\left(X-\mu \right)-\frac{1}{2}{\left(X-\mu \right)}^{T}\Sigma^{-1}\left(-\frac{\partial \mu }{\partial \theta^{i}}\right)$$

And because Σ is symmetric, we also have

$$\frac{\partial }{\partial \theta^{i}}\ln p\left(x|\theta \right)={\left(\frac{\partial \mu }{\partial \theta^{i}}\right)}^{T}\Sigma^{-1}\left(X-\mu \right)$$

As we have $\Sigma =E_{\theta }\left[\left(X-\mu \right){\left(X-\mu \right)}^{T}\right]$, we can write in Equation (1)

$$\begin{array}{cc} g_{ij} & =E_{\theta }\left[{\left(\frac{\partial \mu }{\partial \theta^{i}}\right)}^{T}\Sigma^{-1}\left(X-\mu \right){\left(X-\mu \right)}^{T}\Sigma^{-1}\left(\frac{\partial \mu }{\partial \theta^{j}}\right)\right]\\ \; & ={\left(\frac{\partial \mu }{\partial \theta^{i}}\right)}^{T}\Sigma^{-1}E_{\theta }\left[\left(X-\mu \right){\left(X-\mu \right)}^{T}\right]\Sigma^{-1}\left(\frac{\partial \mu }{\partial \theta^{j}}\right)\\ \; & ={\left(\frac{\partial \mu }{\partial \theta^{i}}\right)}^{T}\Sigma^{-1}\Sigma \Sigma^{-1}\left(\frac{\partial \mu }{\partial \theta^{j}}\right)\\ \; & =\frac{\partial \mu^{T}}{\partial \theta^{i}}\Sigma^{-1}\frac{\partial \mu }{\partial \theta^{j}} \end{array}$$

As θ=μ, we have $\frac{\partial \mu }{\partial \theta^{i}}=e_{i}$, where all the components of $e_{i}$ are zero except the ith component, which is equal to one:

$$\left\{\begin{array}{c} e_{i}^{j}=0,\;i\neq j\\ e_{i}^{j}=1,\;i=j \end{array}\right.$$

and thus

$$g_{ij}=e_{i}^{T}\Sigma^{-1}e_{j}$$

This means that $G=\Sigma^{-1}$, and the partial derivatives are zero: $\frac{\partial g_{ij}}{\partial \theta^{k}}=0$.

And due to Equation (2), we have

$$\forall \;m,i,j=1,\ldots ,n\;\;\;\;\Gamma_{ij,m}:=0$$

This leads to the following transformation of Equation (3):

$$\forall \;m=1,\ldots ,n\;\;\;g_{mk}\frac{d^{2}\mu^{k}}{d\varsigma^{2}}=0$$

This last equation is equivalent to (16). □

In this scenario, assuming Equation (16), the Fisher–Rao distance reduces to the Mahalanobis distance between the means of the multivariate normal distributions [6]; that is, when we consider the distribution N(μ,Σ) with the **Σ** constant, and when we have θ=μ, we can write

(17) $$d\left(\theta_{1},\theta_{2}\right)=d\left(\mu_{1},\mu_{2}\right)=\sqrt{{\left(\mu_{2}-\mu_{1}\right)}^{T}\Sigma^{-1}\left(\mu_{2}-\mu_{1}\right)}$$

**Proof.**  From Equation (16), we deduce that $\frac{d\mu }{d\varsigma }$ is a constant vector:

$$\frac{d\mu }{d\varsigma }=\mu_{2}-\mu_{1}$$

and by replacing it in Equation (4) we get

$$\begin{array}{cc} d(\mu_{1},\mu_{2}) & =\int_{0}^{1}\sqrt{\frac{d\mu^{T}}{d\varsigma }G\frac{d\mu }{d\varsigma }}d\varsigma \\ \; & =\int_{0}^{1}\sqrt{{\left(\mu_{2}-\mu_{1}\right)}^{T}\Sigma^{-1}\left(\mu_{2}-\mu_{1}\right)}d\varsigma \\ \; & =\sqrt{{\left(\mu_{2}-\mu_{1}\right)}^{T}\Sigma^{-1}\left(\mu_{2}-\mu_{1}\right)} \end{array}$$

□

For more general cases, the distance generally does not admit an explicit closed form and must be approximately evaluated with numerical techniques such as geodesic shooting or bound approximations [3].

## 4. Signal Parametric Modeling

### 4.1. Observation of a Finite-Energy Signal

The model used to derive the expression of the statistical distribution has already been applied in previous works [10,11,12,14]. We detail here the hypotheses and give the expression for the law of probability of the observations and its dependence on the parameters.

We are interested in applications recording finite-energy signals. As examples, we can consider chirp frequency-modulated (FM) signals.

At the sensor level, the received signal $s_{r}\left(t\right)$ is a real signal with finite energy, depending on the time *t*, for which we have $\int_{-\infty }^{+\infty }{\left(s_{r}\left(t\right)\right)}^{2}dt<\infty$. This signal admits a Fourier expansion ${\hat{s}}_{r}\left(\nu \right)$, depending on the frequency *ν*. In the real world, the signal is only observed during a limited time interval—t∈[0,T]—and most of the time, it has a finite duration.

We examine the observations after sampling and applying the Fourier transform. Because, for real signals, the Fourier transform values at negative frequencies are the complex conjugates of the Fourier transform values at positive frequencies, we limited the observations to the positive frequencies. The sensor has a limited bandwidth $\overset{ˇ}{B}$, which is supposed to be a continuous interval in $R^{+}$. Due to the limited frequency bandwidth $\overset{ˇ}{B}$ of the sensor, time sampling effect and discrete Fourier transform (DFT), the observation bandwidth is limited to a subset B of positive frequencies: $B\subset \overset{ˇ}{B}\subset R^{+}$. In practice, this subset B is made up of $N_{B}$ regularly spaced discrete values $B=\{\nu_{1},\nu_{2},\ldots \nu_{N_{B}}\}$. After time sampling and the DFT, and from the receiver point of view, the signal is parameterized by a real vector θ, and for frequency dependence, we note that $s_{\theta }\left(\nu \right)$.

As a consequence of Parseval’s theorem, we can write $\sum_{\nu \in B}{\left|s_{\theta }\left(\nu \right)\right|}^{2}\delta \nu <\infty$ (with $\delta \nu =B/N_{B}$, where *B* is the length of the interval $\overset{ˇ}{B}$).

In addition to the above hypotheses, we assume that the observation is composed of the signal mixed with additive stationary random noise. Thus, the observation takes the form.

(18) $$\forall \nu \in B\;x\left(\nu \right)=s_{\theta }\left(\nu \right)+n\left(\nu \right)$$

In this equation, x(ν), $s_{\theta }\left(\nu \right)$ and n(ν) are complex numbers. The total vector of observation x is made up of all complex variables x(ν) in the observation bandwidth: $x={\left(x(\nu )\right)}_{\nu \in B}$. This vector has a total of $N_{B}$ complex coordinates, each corresponding to one of the frequency values in the bandwidth $B=\{\nu_{1},\nu_{2},\ldots \nu_{N_{B}}\}$.

The signal is assumed to be deterministic, which means that each value of the parameter vector θ corresponds to a single frequency function $\nu \mapsto s_{\theta }\left(\nu \right)$.

Looking at the noise contributions in the frequency domain n(ν), we assume that the noise components after the Fourier transform n(ν) are centered, circularly symmetric complex Gaussian random variables which are independent across frequencies. The Gaussianity is true if the noise is naturally Gaussian, but it is also asymptotically true for other types of noise distributions. The asymptotic independence for any pair of frequencies is reported in [9].

We assume that the noise spectral power density in the bandwidth is known and defined by $\forall \nu \in B\;\;\;\gamma_{0}\left(\nu \right)=E\left(n\left(\nu \right)n^{*}\left(\nu \right)\right)$. The hypothesis that the Gaussian complex noise is circularly symmetric means that $E\left(n(\nu )n(\nu )\right)=0$.

When assuming all previous hypotheses, the law of the total vector of the observations $x={\left(x(\nu )\right)}_{\nu \in B}$ can be expressed as follows:

(19) $$p\left(x|\theta \right)=\prod_{\nu \in B}\frac{1}{\pi \gamma_{0}\left(\nu \right)}\exp\left(-\frac{{\left|x\left(\nu \right)-s_{\theta }\left(\nu \right)\right|}^{2}}{\gamma_{0}\left(\nu \right)}\right)$$

The total vector of observation x has a total number $N_{B}$ of complex components and can be rewritten as a real vector X with $2N_{B}$ real components. With this notation, Equation (19) is similar to the normal multivariate distribution expressed in [6] and in Equation (5):

(20) $$p\left(X|\theta \right)=\frac{1}{2^{N_{B}}\pi^{N_{B}}\sqrt{\det\left(\Sigma \right)}}\exp\left(-\frac{{\left(X-\mu_{\theta }\right)}^{T}\Sigma_{0}^{-1}\left(X-\mu_{\theta }\right)}{2}\right)$$

where the following definitions apply:

- $N_{B}$ is the number of frequencies *ν* in B;
- $\Sigma_{0}=diag\left(\sigma_{0}^{2}\left(n\right)\right)$ is a diagonal $2N_{B}\times 2N_{B}$ matrix such that
  $$\forall k=1,\ldots N_{B}\;\;\;\sigma_{0}^{2}\left(2k-1\right)=\sigma_{0}^{2}\left(2k\right)=\frac{\gamma_{0}\left(\nu_{k}\right)}{2},$$
- X is a $2N_{B}$ vector, with $\forall k=1,\ldots N_{B}\;\;X^{2k-1}=Re\left\{x(k)\right\}$ and $X^{2k}=Im\left\{x(k)\right\}$;
- $\mu_{\theta }$ is a $2N_{B}$ vector, with $\forall k=1,\ldots N_{B}\;\;\mu_{\theta }^{2k-1}=Re\left\{s_{\theta }\left(k\right)\right\}$ and $\mu_{\theta }^{2k}=Im\left\{s_{\theta }\left(k\right)\right\}$.

### 4.2. Signal Dependence on Parameters and the Manifolds L2(B,θ)

Equation (19) (or equivalently Equation (20)) defines a family of probability distributions $S_{\Theta }$ when the parameter θ varies in a predefined set Θ:

(21) $$S_{\Theta }=\left\{p_{\theta }=p\left(x|\theta \right)\;|\;\theta \in \Theta \right\}$$

With additional regularity conditions, $S_{\Theta }$ can be considered a statistical manifold (see [9,12], Section 2.1 for more details).

As a consequence, the geometry of the manifold lies in Equation (19) (or in Equation (20)) and in the definition of the parameter set Θ. We call such a manifold manifold L2(B,θ). As θ has *N* components, with $N\leq 2N_{B}$, *N* is the dimension of the manifold L2(B,θ).

For all ν in B, the signal $s_{\theta }\left(\nu \right)$ is a complex value, which can be represented by a modulus (or magnitude) $\rho_{\theta }\left(\nu \right)$ and a phase $\psi_{\theta }\left(\nu \right)$:

(22) $$s_{\theta }\left(\nu \right)=\rho_{\theta }\left(\nu \right)\cdot \exp\left(ı\psi_{\theta }\left(\nu \right)\right)$$

with $\psi_{\theta }\left(\nu \right)\in ]-\pi ,\pi ]$ and $\rho_{\theta }\left(\nu \right)\in ]0,+\infty [$.

As explained in [12], the parameters θ of the signal can be split into two parts: $\theta =\left(\begin{array}{c} \phi \\ \varphi \end{array}\right)$. One part, ϕ, with *P* components, is related to the magnitude $\rho_{\phi }\left(\nu \right)$ of the signal, and the other part, φ, with N−P components, is related to the phase $\psi_{\varphi }\left(\nu \right)$ of the signal:

(23) $$\forall \nu \in B\;\;\;\;\;s_{\theta }\left(\nu \right)=\rho_{\phi }\left(\nu \right)\cdot \exp\left(ı\psi_{\varphi }\left(\nu \right)\right)$$

In the following, any manifold L2(B,θ) is described by Equation (19) completed with Equation (23), which refers to the case where $\theta =\left(\begin{array}{c} \phi \\ \varphi \end{array}\right)$.

As the magnitudes and phases can be determined separately from the $s_{\theta }\left(\nu \right)$, we assume that the numbers of coordinates of the parameter vectors θ verify the following properties:

(24) $$\left\{\begin{array}{c} P\leq N_{B}\\ N-P\leq N_{B} \end{array}\right.$$

In a manifold L2(B,θ), the expression of the distribution is given by

(25) $$p\left(x|\theta \right)=\prod_{\nu \in B}\frac{1}{\pi \gamma_{0}\left(\nu \right)}\exp\left(-\frac{{\left|x\left(\nu \right)-\rho_{\phi }\left(\nu \right)\cdot \exp\left(ı\psi_{\varphi }\left(\nu \right)\right)\right|}^{2}}{\gamma_{0}\left(\nu \right)}\right)$$

More details regarding the regularity conditions and dependence of the phase $\psi_{\varphi }\left(\nu \right)$ on the frequency *ν* are available in [12].

## 5. General Expressions of the Fisher–Rao Distances on the Manifolds L2(B,θ)

### 5.1. Geodesic Equations on the Manifolds L2(B,θ)

To compute the geodesic equations on a manifold L2(B,θ), we use the method described in Section 2 and Equations (1)–(3) and (25). We obtained the following theorem already expressed in [10,11,12].

**Theorem 1**  (Linearly dependant gradient (LDG) theorem)**.** *The (Fisher-)geodesic equations in the submanifold L2(B,θ) reduce to the following two vectorial equations:*

(26) $$\left\{\begin{array}{c} \sum_{\nu \in B}\frac{2{\left(\rho_{\theta }\left(\nu \right)\right)}^{2}}{\gamma_{0}\left(\nu \right)}\left(\frac{d^{2}\ln\rho_{\theta }}{d\varsigma^{2}}\left(\nu \right)+{\left(\frac{d\ln\rho_{\theta }}{d\varsigma }\left(\nu \right)\right)}^{2}-{\left(\frac{d\psi_{\theta }}{d\varsigma }\left(\nu \right)\right)}^{2}\right){▽}_{\theta }\ln\rho_{\theta }\left(\nu \right)={\left[0\right]}_{N}\\ \sum_{\nu \in B}\frac{2{\left(\rho_{\theta }\left(\nu \right)\right)}^{2}}{\gamma_{0}\left(\nu \right)}\left(\frac{d^{2}\psi_{\theta }}{d\varsigma^{2}}\left(\nu \right)+2\frac{d\ln\rho_{\theta }}{d\varsigma }\left(\nu \right)\frac{d\psi_{\theta }}{d\varsigma }\left(\nu \right)\right){▽}_{\theta }\psi_{\theta }\left(\nu \right)={\left[0\right]}_{N} \end{array}\right.$$

**Proof.**  The method explained in Section 2.2 consists of three steps:

- Calculate the Fisher information matrix ***G***.
- Calculate the partial derivatives $\partial_{i}G$ and the Christoffel symbols $\Gamma_{ij,k}$.
- Replace the Christoffel symbols to express the geodesic equation.

The calculations are detailed in [12].

The first step consists of calculating the Fisher information matrix.

It can be shown that the Fisher matrix is a block diagonal matrix:

$$G=\left[g_{\mathrm{ij}}\right]=\left(\begin{array}{cc} \left[g_{uv}\right] & \left[0\right]\\ \left[0\right] & \left[g_{qr}\right] \end{array}\right)$$

The second step consists of calculating the Christoffel symbols, based on the partial derivative of the Fisher matrix coefficient with respect to the parameters. In the third step of the proof, we replace the values of $\Gamma_{ij,m}$ in the geodesic equation (Equation (3)) in order to obtain geodesic equations expressed on the manifolds L2(B,θ).

Finally, we can write the geodesic equations as follows:

(27) $$\left\{\begin{array}{c} \sum_{\nu \in B}\frac{2}{\gamma_{0}\left(\nu \right)}\left(\frac{d^{2}\rho_{\phi }}{d\varsigma^{2}}\left(\nu \right)-\rho_{\phi }\left(\nu \right){\left(\frac{d\psi_{\varphi }}{d\varsigma }\left(\nu \right)\right)}^{2}\right){▽}_{\phi }\rho_{\phi }\left(\nu \right)={\left[0\right]}_{P}\\ \sum_{\nu \in B}\frac{2}{\gamma_{0}\left(\nu \right)}\left(\frac{d^{2}\psi_{\varphi }}{d\varsigma^{2}}\left(\nu \right)+2\rho_{\phi }\left(\nu \right)\frac{d\rho_{\phi }}{d\varsigma }\left(\nu \right)\frac{d\psi_{\varphi }}{d\varsigma }\left(\nu \right)\right){▽}_{\varphi }\psi_{\varphi }\left(\nu \right)={\left[0\right]}_{N-P} \end{array}\right.$$

Equation (27) can easily be transformed into Equation (26). □

### 5.2. Fisher–Rao Distances on the Manifolds L2(B,θ)

**Theorem 2.**  *With ${\tilde{\rho }}_{\theta }$ and ${\tilde{\psi }}_{\theta }$ as solutions of the geodesic equations, the Fisher–Rao distance on the submanifold L2(B,θ) can be calculated as the following integral:*

(28) $$d_{L2,B,\theta }\left(\theta_{1},\theta_{2}\right)=\int_{0}^{1}\sqrt{\sum_{\nu \in B}\frac{2{\left({\tilde{\rho }}_{\theta }\left(\nu \right)\right)}^{2}}{\gamma_{0}\left(\nu \right)}\left({\left(\frac{d\ln{\tilde{\rho }}_{\theta }}{d\varsigma }\left(\nu \right)\right)}^{2}+{\left(\frac{d{\tilde{\psi }}_{\theta }}{d\varsigma }\left(\nu \right)\right)}^{2}\right)}d\varsigma$$

**Proof.**  Detailed calculations are available in [12].

We note that ${\tilde{\phi }}^{u}$ (or ${\tilde{\phi }}^{v}$) and ${\tilde{\varphi }}^{q}$ (or ${\tilde{\varphi }}^{r}$) are the solutions of the geodesic equation (Equation (27)). They are expressed as functions of ς. From Equation (4), taking into account the block diagonal structure of the Fisher information matrix as demonstrated in Section 5.1, we derive the Fisher–Rao distance from the following integral:

(29) $$d_{L2,B,\theta }\left(\theta_{1},\theta_{2}\right)=\int_{0}^{1}\sqrt{g_{uv}\frac{d{\tilde{\phi }}^{u}}{d\varsigma }\frac{d{\tilde{\phi }}^{v}}{d\varsigma }+g_{qr}\frac{d{\tilde{\varphi }}^{q}}{d\varsigma }\frac{d{\tilde{\varphi }}^{r}}{d\varsigma }}d\varsigma$$

We replace the $g_{uv}$ and $g_{qr}$ with their expressions and we use the following equalities:

$$\partial_{u}\rho_{\phi }\left(\nu \right)\cdot \frac{d\phi^{u}}{d\varsigma }=\frac{\partial \rho_{\phi }}{\partial \phi^{u}}\left(\nu \right)\frac{d\phi^{u}}{d\varsigma }=\frac{d\rho_{\phi }}{d\varsigma }\left(\nu \right)$$

$$\partial_{r}\psi_{\varphi }\left(\nu \right)\cdot \frac{d\varphi^{r}}{d\varsigma }=\frac{\partial \psi_{\varphi }}{\partial \varphi^{r}}\left(\nu \right)\frac{d\varphi^{r}}{d\varsigma }=\frac{d\psi_{\varphi }}{d\varsigma }\left(\nu \right)$$

We then obtain

(30) $$d_{L2,B,\theta }\left(\theta_{1},\theta_{2}\right)=\int_{0}^{1}\sqrt{\sum_{\nu \in B}\frac{2}{\gamma_{0}\left(\nu \right)}\left({\left(\frac{d{\tilde{\rho }}_{\phi }}{d\varsigma }\left(\nu \right)\right)}^{2}+{\left({\tilde{\rho }}_{\phi }\left(\nu \right)\frac{d{\tilde{\psi }}_{\varphi }}{d\varsigma }\left(\nu \right)\right)}^{2}\right)}d\varsigma$$

By definition, ${\tilde{\rho }}_{\theta }={\tilde{\rho }}_{\phi }$, and ${\tilde{\psi }}_{\theta }={\tilde{\psi }}_{\varphi }$.

Due to the derivative properties, we have $\frac{1}{{\tilde{\rho }}_{\theta }\left(\nu \right)}\frac{d{\tilde{\rho }}_{\theta }}{d\varsigma }\left(\nu \right)=\frac{d\ln{\tilde{\rho }}_{\theta }}{d\varsigma }\left(\nu \right)$.

Using these equalities, Equation (30) is easily transformed into Equation (28). □

To compute the expression in Equation (28) and give a closed-form expression of the Fisher–Rao distance, it is necessary to solve Equation (26). This can only be accomplished when the parametric expressions of a magnitude $\rho_{\theta }\left(\nu \right)$ and phase $\psi_{\theta }\left(\nu \right)$ are expressed in closed forms, i.e., when the model is particularized. This is performed with the specific manifold in the next section.

## 6. General Expressions of the Fisher–Rao Distances on the Manifold L2(B)

### 6.1. The Finite-Energy Signals and the Manifold L2(B)

Here, we look at a specific manifold corresponding to the situation for which, in Equation (20), the parameters are the components of the vector $\mu_{\theta }$. This means that $\mu_{\theta }=\theta$.

In this case, θ is a vector with $2N_{B}$ components, and $\forall k=1,\ldots 2N_{B}\;\;\;\theta^{k}=\mu^{k}$. In other words, $\Theta =R^{2N_{B}}$. This means that all the signal components $s_{\theta }\left(\nu \right)$ vary freely in C.

Variations in all signal components determine a manifold, which we call the manifold L2(B). This corresponds to all signals with finite energy observed in the frequency bandwidth B. The dimension of the manifold L2(B) is $2N_{B}$. The manifold L2(B) corresponds exactly to the manifold described by $2N_{B}$ multivariate normal distributions with the same diagonal covariance matrix $\Sigma_{0}$ and different mean values.

Any manifold L2(B,θ) is a sub-manifold of the manifold L2(B).

### 6.2. Geodesic Equations on the Manifold L2(B)

The geodesic equation on the manifold L2(B) is derived directly from Equations (6) and (20) when the coefficients of the matrix $\Sigma_{0}$ are not part of the coordinates. In this case, the second line of Equation (6) does not exist, and the geodesic equation becomes

(31) $$\left\{\begin{array}{c} \frac{d^{2}\mu_{\theta }}{d\varsigma^{2}}={\left[0\right]}_{N} \end{array}\right.$$

The solution between $\mu_{\theta_{1}}$ and $\mu_{\theta_{2}}$ is an affine function of ς [6]:

(32) $$\forall \varsigma \in \left[0,1\right]\;\;\;\;{\tilde{\mu }}_{\theta }\left(\varsigma \right)=\mu_{\theta_{1}}+\varsigma \left(\mu_{\theta_{2}}-\mu_{\theta_{1}}\right)$$

### 6.3. Fisher–Rao Distance on the Manifold L2(B)

From Equation (20) and the definition of the Fisher matrix in Equation (1), we can easily derive the expression of the Fisher information matrix $G={\left[g_{\mathrm{ij}}\right]}_{N\times N}$ in the manifold L2(B):

(33) $$g_{ij}=\frac{\partial \mu_{\theta }^{T}}{\partial \theta^{i}}\Sigma_{0}^{-1}\frac{\partial \mu_{\theta }}{\partial \theta^{j}}$$

If we report this expression of G in Equation (4), then we get the expression of the Fisher–Rao distance on the manifold L2(B):

(34) $$d_{L2,B}\left(\theta_{1},\theta_{2}\right)=\int_{0}^{1}\sqrt{\frac{d{\tilde{\mu }}_{\theta }^{T}}{d\varsigma }\Sigma_{0}^{-1}\frac{d{\tilde{\mu }}_{\theta }}{d\varsigma }}d\varsigma$$

It is known from [6] that in the $2N_{B}$-dimensional manifold composed of multivariate normal distributions with a common covariance matrix $\Sigma_{0}$, the Fisher–Rao distance between two distributions parametrised by $\mu_{\theta_{1}}$ and $\mu_{\theta_{2}}$ is equal to the Mahalanobis distance. This can be easily verified here. When changing $\tilde{\mu }$ in Equation (34) by its value from Equation (32), we have

(35) $$d_{L2,B}\left(\theta_{1},\theta_{2}\right)=\sqrt{{\left(\mu_{\theta_{2}}-\mu_{\theta_{1}}\right)}^{T}\Sigma_{0}^{-1}\left(\mu_{\theta_{2}}-\mu_{\theta_{1}}\right)}$$

With the notation of Equations (20) and (22), this Fisher–Rao distance on the manifold L2(B) can be rewritten as follows:

(36) $$\begin{array}{cc} \; & d_{L2,B}\left(\theta_{1},\theta_{2}\right)\\ \; & =\sqrt{\sum_{\nu \in B}\frac{2}{\gamma_{0}\left(\nu \right)}\left({\left(\rho_{\theta_{2}}\left(\nu \right)\right)}^{2}+{\left(\rho_{\theta_{1}}\left(\nu \right)\right)}^{2}-2\rho_{\theta_{2}}\left(\nu \right)\cdot \rho_{\theta_{1}}\left(\nu \right)\cdot \cos\left(\Delta \psi (\nu )\right)\right)} \end{array}$$

with

(37) $$\Delta \psi \left(\nu \right)=\psi_{\theta_{2}}\left(\nu \right)-\psi_{\theta_{1}}\left(\nu \right)$$

**Proof.**  The $N_{B}$ frequencies in B are $\nu_{1}$, $\nu_{2}$, …, $\nu_{N_{B}}$.

$\Sigma_{0}$ is a diagonal $2N_{B}\times 2N_{B}$ real matrix. Each value $\nu_{i}$ in the frequency band is represented by two diagonal values, ${\left(\sigma_{0}\left(2i-1\right)\right)}^{2}$ and ${\left(\sigma_{0}\left(2i\right)\right)}^{2}$, and we have by definition

$${\left(\sigma_{0}\left(2i-1\right)\right)}^{2}={\left(\sigma_{0}\left(2i\right)\right)}^{2}=\frac{\gamma (\nu_{i})}{2}$$

The vector $\mu_{\theta }$ has $2N_{B}$ components arranged in pairs:

$$\left\{\begin{array}{c} \mu_{\theta }^{2i-1}=Re\left\{s_{\theta }\left(\nu_{i}\right)\right\}=\rho_{\theta }\left(\nu_{i}\right)\cdot \cos\left(\psi_{\theta }\left(\nu_{i}\right)\right)\\ \mu_{\theta }^{2i}=Im\left\{s_{\theta }\left(\nu_{i}\right)\right\}=\rho_{\theta }\left(\nu_{i}\right)\cdot \sin\left(\psi_{\theta }\left(\nu_{i}\right)\right) \end{array}\right.$$

At frequency $\nu_{i}$, the contribution of the components indexed by 2i−1 and 2i is as follows:

$$\begin{array}{cc} \; & \frac{{\left(\mu_{2}^{2i-1}-\mu_{1}^{2i-1}\right)}^{2}}{{\left(\sigma_{0}\left(2i-1\right)\right)}^{2}}+\frac{{\left(\mu_{2}^{2i}-\mu_{1}^{2i}\right)}^{2}}{{\left(\sigma_{0}\left(2i\right)\right)}^{2}}\\ \; & =\frac{2}{\gamma (\nu_{i})}\cdot \left({\left(\rho_{\theta_{2}}\left(\nu_{i}\right)\cos\left(\psi_{\theta_{2}}\left(\nu_{i}\right)\right)-\rho_{\theta_{1}}\left(\nu_{i}\right)\cos\left(\psi_{\theta_{1}}\left(\nu_{i}\right)\right)\right)}^{2}\right)\\ \; & +\frac{2}{\gamma (\nu_{i})}\cdot \left({\left(\rho_{\theta_{2}}\left(\nu_{i}\right)\sin\left(\psi_{\theta_{2}}\left(\nu_{i}\right)\right)-\rho_{\theta_{1}}\left(\nu_{i}\right)\sin\left(\psi_{\theta_{1}}\left(\nu_{i}\right)\right)\right)}^{2}\right)\\ \; & =\frac{2}{\gamma (\nu_{i})}\cdot \left({\left(\rho_{\theta_{2}}\left(\nu_{i}\right)\right)}^{2}+{\left(\rho_{\theta_{1}}\left(\nu_{i}\right)\right)}^{2}\right)\\ \; & -\frac{2}{\gamma (\nu_{i})}\cdot 2\rho_{2}\left(\nu_{i}\right)\rho_{1}\left(\nu_{i}\right)\cdot \left(\cos\left(\psi_{2}\left(\nu_{i}\right)\right)\cos\left(\psi_{1}\left(\nu_{i}\right)\right)+\sin\left(\psi_{\theta_{2}}\left(\nu_{i}\right)\right)\sin\left(\psi_{\theta_{1}}\left(\nu_{i}\right)\right)\right)\\ \; & =\frac{2}{\gamma (\nu_{i})}\cdot \left({\left(\rho_{\theta_{2}}\left(\nu_{i}\right)\right)}^{2}+{\left(\rho_{\theta_{1}}\left(\nu_{i}\right)\right)}^{2}\right)-\frac{2}{\gamma (\nu_{i})}\cdot 2\rho_{\theta_{2}}\left(\nu_{i}\right)\rho_{\theta_{1}}\left(\nu_{i}\right)\cos\left(\psi_{\theta_{2}}\left(\nu_{i}\right)-\psi_{\theta_{1}}\left(\nu_{i}\right)\right)\\ \; & =\frac{2}{\gamma (\nu_{i})}\cdot \left({\left(\rho_{\theta_{2}}\left(\nu_{i}\right)\right)}^{2}+{\left(\rho_{\theta_{1}}\left(\nu_{i}\right)\right)}^{2}-2\rho_{\theta_{2}}\left(\nu_{i}\right)\rho_{\theta_{1}}\left(\nu_{i}\right)\cos\left(\psi_{\theta_{2}}\left(\nu_{i}\right)-\psi_{\theta_{1}}\left(\nu_{i}\right)\right)\right) \end{array}$$

□

### 6.4. Expression with the Spectral Distance Function

We define the spectral distance function as follows:

(38) $$\forall \nu \in B\;\;\;f_{\theta_{1},\theta_{2}}\left(\nu \right)=\sqrt{\frac{2}{\gamma_{0}\left(\nu \right)}\left({\left(\rho_{\theta_{2}}\left(\nu \right)\right)}^{2}+{\left(\rho_{\theta_{1}}\left(\nu \right)\right)}^{2}-2\rho_{\theta_{2}}\left(\nu \right)\cdot \rho_{\theta_{1}}\left(\nu \right)\cdot \cos\left(\Delta \psi (\nu )\right)\right)}$$

This function describes the contribution of each spectral component of the signal observations $\theta_{1}$ and $\theta_{2}$ to their Fisher–Rao distance.

According to Equation (36), the Fisher–Rao distance can be expressed from the spectral distance function by

(39) $$d_{L2,B}\left(\theta_{1},\theta_{2}\right)=\sqrt{\sum_{\nu \in B}{\left(f_{\theta_{1},\theta_{2}}\left(\nu \right)\right)}^{2}}$$

When the signals have a continuous spectrum, this expression takes the form

(40) $$d_{L2,B}\left(\theta_{1},\theta_{2}\right)=\sqrt{\frac{1}{B}\int_{\nu \in \overset{ˇ}{B}}{\left(f_{\theta_{1},\theta_{2}}\left(\nu \right)\right)}^{2}d\nu }$$

## 7. Evaluation of the Fisher–Rao Distances

### 7.1. Comparison of the Fisher–Rao Distances on the Manifold L2(B) and on Any Sub-Manifold L2(B,θ)

The *N*-dimensional sub-manifolds L2(B,θ) are included in the $2N_{B}$-dimensional manifold L2(B) composed by multivariate normal distributions with a common diagonal covariance matrix $\Sigma_{0}$. The metric on any sub-manifold L2(B,θ) is the induced metric from the ambient manifold L2(B). When two endpoints are connected with a geodesic path on the sub-manifold, they are also connected with the same path on the ambient manifold. However, this path on the ambient manifold is not always the minimum (geodesic) path for the ambient manifold. As a consequence, we expect that the Fisher–Rao distance $d_{L2,B,\theta }\left(\theta_{1},\theta_{2}\right)$ measured on any sub-manifold L2(B,θ) is greater than or equal to the Fisher–Rao distance measured on the manifold L2(B):

(41) $$d_{L2,B,\theta }\left(\theta_{1},\theta_{2}\right)\geq d_{L2,B}\left(\theta_{1},\theta_{2}\right)$$

When both quantities are always equal, the sub-manifold L2(B,θ) is said to be fully geodesic on the manifold L2(B). As a consequence of Equation (41), the value of the Fisher–Rao distance between two finite-energy signals on the manifold L2(B) is a minimum bound for any Fisher–Rao distance between both finite-energy signals calculated on any sub-manifold L2(B,θ).

### 7.2. Asymptotic Behavior of the Fisher–Rao Distances

**Theorem 3.**  *The Fisher-Rao distances belong to the following interval:*

(42) $$\sqrt{\sum_{\nu \in B}\frac{2{\left(\rho_{\theta_{2}}\left(\nu \right)-\rho_{\theta_{1}}\left(\nu \right)\right)}^{2}}{\gamma_{0}\left(\nu \right)}}\leq d_{L2,B}\left(\theta_{1},\theta_{2}\right)\leq \sqrt{\sum_{\nu \in B}\frac{2{\left(\rho_{\theta_{2}}\left(\nu \right)+\rho_{\theta_{1}}\left(\nu \right)\right)}^{2}}{\gamma_{0}\left(\nu \right)}}$$

**Proof.**  The value of $\cos\left(\Delta \psi (\nu )\right)$ in Equation (36) varies between −1 and +1. □

**Theorem 4.**  *When the phase differences Δψ(ν) are small, distances tend toward the minimum values:*

(43) $$d_{L2,B}\left(\theta_{1},\theta_{2}\right)\underset{\Delta \psi (\nu )\to 0}{⟶}\sqrt{\sum_{\nu \in B}\frac{2{\left(\rho_{\theta_{2}}\left(\nu \right)-\rho_{\theta_{1}}\left(\nu \right)\right)}^{2}}{\gamma_{0}\left(\nu \right)}}$$

**Proof.**  The value of $\cos\left(\Delta \psi (\nu )\right)$ tends toward one in Equation (36). □

**Theorem 5.**  *When the signal at $\theta_{1}$ and the signal $\theta_{2}$ have separate bandwidths, or when the phase differences vary significantly across the bandwidth, the Fisher–Rao distance becomes*

(44) $$d_{L2,B}\left(\theta_{1},\theta_{2}\right)\approx \sqrt{SNR_{1}+SNR_{2}}$$

*With:*

(45) $$\begin{array}{cc} SNR_{1} & =\sum_{\nu \in B}\frac{2{\left(\rho_{\theta_{1}}\left(\nu \right)\right)}^{2}}{\gamma_{0}\left(\nu \right)} \end{array}$$

(46) $$\begin{array}{cc} SNR_{2} & =\sum_{\nu \in B}\frac{2{\left(\rho_{\theta_{2}}\left(\nu \right)\right)}^{2}}{\gamma_{0}\left(\nu \right)} \end{array}$$

**Proof.**  The value of $\sum_{\nu \in B}\frac{2\rho_{\theta_{1}}\left(\nu \right)\rho_{\theta_{2}}\left(\nu \right)}{\gamma_{0}\left(\nu \right)}\cos\left(\Delta \psi (\nu )\right)$ tends toward zero in Equation (36). □

In this expressions, $SNR_{1}$ and $SNR_{2}$ represent the signal-to-noise ratios at $\theta_{1}$ and $\theta_{2}$, respectively.

### 7.3. Numerical Application

We see with Equation (36) that the Fisher–Rao distance is proportional to the square root of the signal-to-noise ratio (*SNR*), meaning that when the signal-to-noise ratios $SNR_{1}$ and $SNR_{2}$ are multiplied by *a*, the Fisher–Rao distance is multiplied by $\sqrt{a}$ (if the phase differences remain the same).

Furthermore, we observe that the Fisher–Rao distance is symmetric under the permutations of $\theta_{1}$ and $\theta_{2}$.

As a consequence, we can explore the distance at a reference signal-to-noise ratio $SNR_{1}=1$ with the hypothesis that $SNR_{2}\leq 1$.

We assume that the noise is Gaussian white with a constant spectral density $\gamma_{0}\left(\nu \right)$.

We examine several signal configurations and their respective distances.

We compare two linear frequency-modulated (LFM) pulses with the same time duration but different bandwidths. The pulses were weighted by a Hann window. The parametric expressions of the signals are as follows:

(47) $$\forall t\in \left[0,T\right]\;\;\;s_{r}\left(t\right)=\left(0.5-0.5*\cos(2\pi \frac{t}{T})\right)\cdot \sin\left(\eta +2\pi \left(\left(f_{0}-\epsilon \frac{B_{FM}}{2}\right)t+\epsilon \frac{B_{FM}}{2T}t^{2}\right)\right)$$

In Equation (47), the meanings of the parameters are as follows:

- $f_{0}$ is the middle frequency of the modulation;
- $B_{FM}$ is the frequency bandwidth of the modulation;
- η is a phase parameter between 0 and 2π which introduces a phase shift;
- ϵ takes the values +1 and −1, indicating whether the slope of the LFM frequency variation is upward or downward, respectively;
- *T* is the time length of the impulsion.

After sampling at frequency $F_{s}$, as we choose *T* to also be the time duration of the Fourier transform, we obtain $N_{T}=F_{s}T$ samples, and following the DFT, the observations become

(48) $$\forall \nu \in B\;\;\;s_{\theta }\left(\nu \right)=\sum_{n=1}^{N_{T}}s_{r}\left(\frac{n-1}{F_{s}}\right)\exp\left(-2ı\pi \frac{\nu }{F_{s}}\left(n-1\right)\right)$$

with the following definitions:

- $B=\{\nu_{1},\nu_{2},\ldots \nu_{N_{B}}\}$;
- $\forall k=1\ldots N_{B}\;\;\;\nu_{k}=\left(k-1\right)\frac{F_{s}}{N_{T}}$;
- If $N_{T}$ is even, then $N_{B}=\frac{N_{T}}{2}+1$, or if $N_{T}$ is odd, then $N_{B}=\frac{N_{T}-1}{2}+1$.

For the numerical applications, we choose the following:

- The time duration is T=1.000 s;
- The sample frequency Fs is fixed to 1.024 Hz;
- This determines $N_{T}=1024$ and $N_{B}=513$.

**Figure 1:**
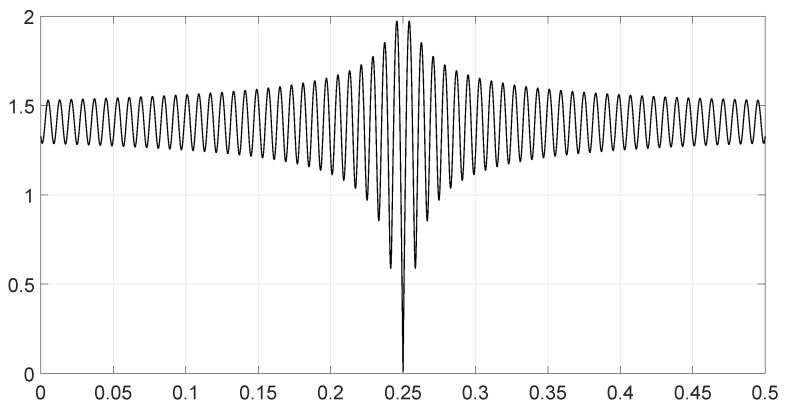
Value of the Fisher–Rao distance as a function of the bandwidth $B_{FM}$ of the signal $\theta_{2}$ for scenario 1.

**Figure 2:**
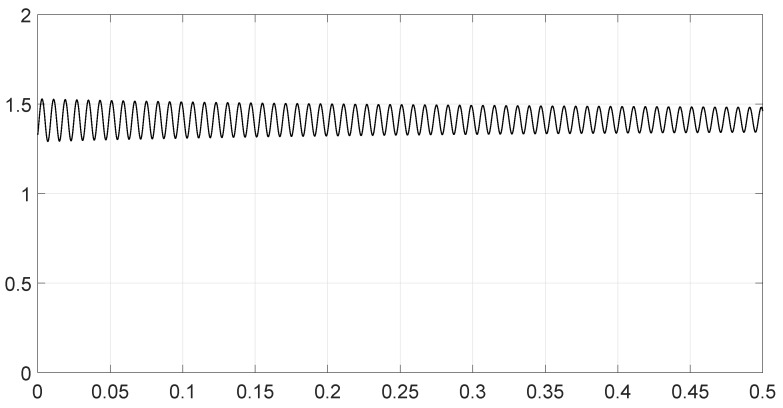
Value of the Fisher–Rao distance as a function of the bandwidth $B_{FM}$ of the signal $\theta_{2}$ for scenario 2.

**Figure 3:**
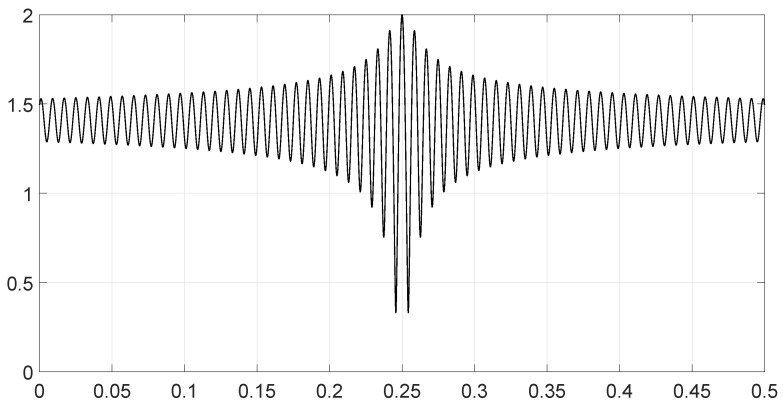
Value of the Fisher–Rao distance as a function of the bandwidth $B_{FM}$ of the signal $\theta_{2}$ for scenario 3.

**Figure 4:**
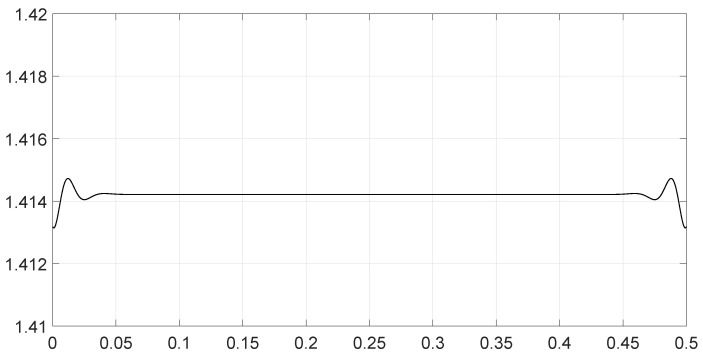
Value of the Fisher–Rao distance as a function of the bandwidth $B_{FM}$ of the signal $\theta_{2}$ for scenario 4.

**Figure 5:**
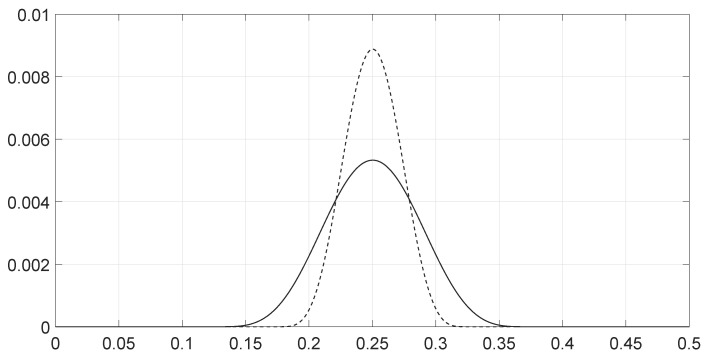
Values of the spectra $\rho_{\theta_{1}}\left(\nu \right)$ (solid line) and $\rho_{\theta_{2}}\left(\nu \right)$ (dashed line) for the two signals as a function of the frequency *ν* for scenario 5.

**Figure 6:**
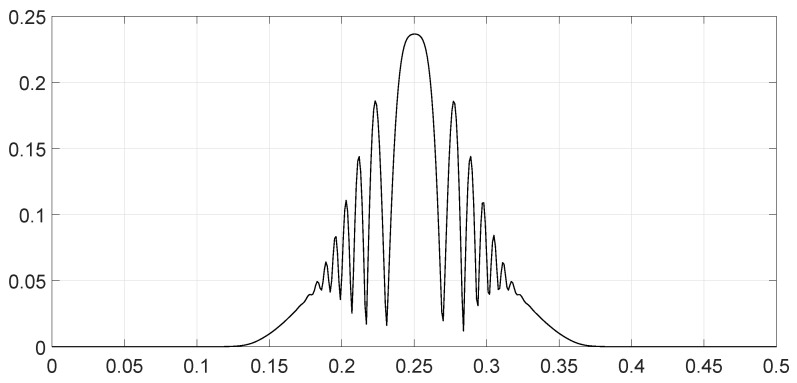
Values of the spectral distance $f_{\theta_{1},\theta_{2}}\left(\nu \right)$ as a function of the frequency ν for scenario 5.

**Figure 7:**
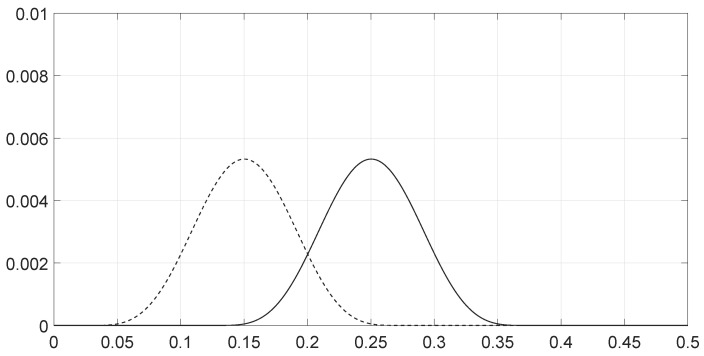
Values of the spectra $\rho_{\theta_{1}}\left(\nu \right)$ (solid line) and $\rho_{\theta_{2}}\left(\nu \right)$ (dashed line) for the two signals as a function of the frequency *ν* for scenario 6.

**Figure 8:**
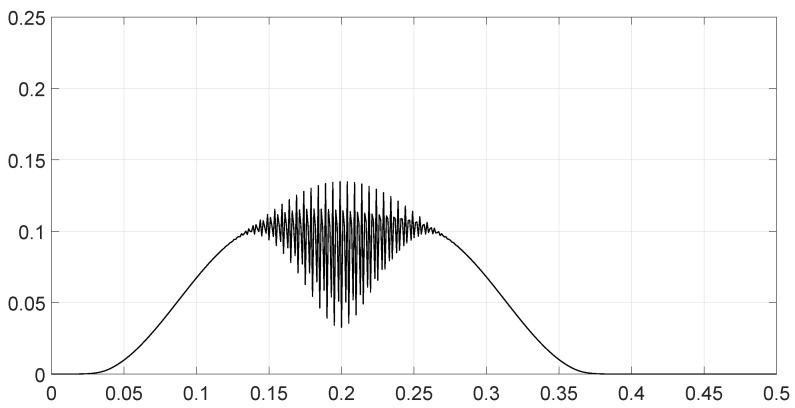
Values of the spectral distance $f_{\theta_{1},\theta_{2}}\left(\nu \right)$ as a function of the frequency *ν* for scenario 6.

**Figure 9:**
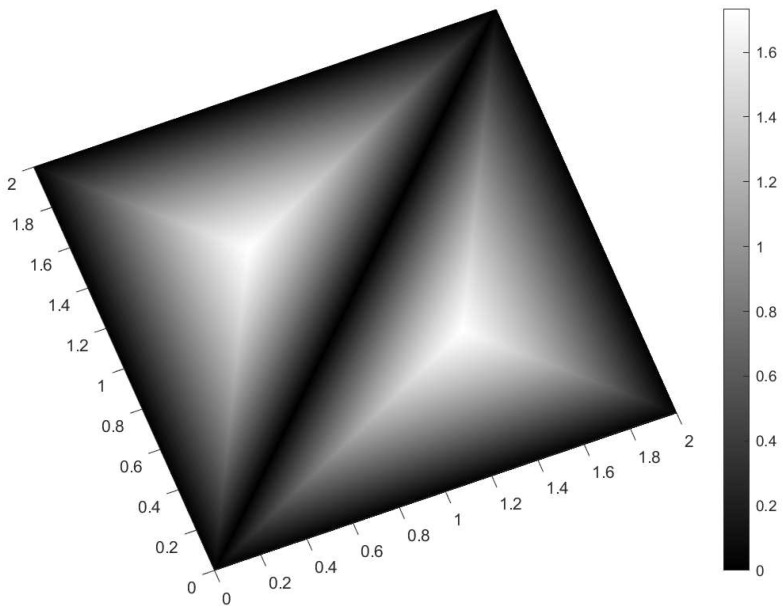
3D plot of $d_{min}$ for the toy problem of scenario 7. The X axis represents $\eta (\theta_{2})$, and the Y axis represents $\eta (\theta_{3})$. The color bar indicates the values of $d_{min}$ (Z axis). The X and Y axes are graduated in *π* values (e.g., 2 means 2*π*, and 0.6 means 0.6*π*). We see that maximum values of $d_{min}$ were reached for the parameter values $\left(\eta \left(\theta_{2}\right),\eta \left(\theta_{3}\right)\right)=\left(2\pi /3,4\pi /3\right)$ or $\left(\eta \left(\theta_{2}\right),\eta \left(\theta_{3}\right)\right)=\left(4\pi /3,2\pi /3\right)$.

We analyzed various scenarios; each was characterized by a set of parameters and illustrated with figures presenting the calculation results:

- Scenario 1: Similar signals with different bandwidths.
  The parameters are described in Table 1. Both signals were similar (same phase and middle frequency and same slope of modulation) except for their respective bandwidths. The figure shows variation in the distance with respect to the bandwidth of signal $\theta_{2}$.
  The results are shown in Figure 1. We observe that th
- Scenario 2: Signals with different bandwidths and slopes of modulation.
  The parameters are described in Table 2. Both signals were similar (same phase and middle frequency) except for their respective bandwidths and their slopes of modulation. The figure shows variation in the distance with respect to the bandwidth of signal $\theta_{2}$.
  The results are shown in Figure 2. We observe that the distance varied slightly around 1.4142 ($\sqrt{2}$). This is a situation close to Equation (44).
- Scenario 3: Signals with opposite phases and different bandwidths.
  The parameters are described in Table 3. Both signals were similar (same phase and middle frequency and same slope of modulation) except for their respective bandwidths, and they had a phase difference of *π*. The figure shows variation in the distance with respect to the bandwidth of signal $\theta_{2}$.
  The results are shown in Figure 3. We observe that the distance varied between 0 and 2. This corresponds to the bounds of Equation (42). The maximum was reached when the two signals were identical with a phase shift of *π* (same bandwidth $B_{FM}$).
- Scenario 4: Signals with different bandwidths and mid frequencies.
  The parameters are described in Table 4. The signals had different middle frequencies, but they had the same phase and the same slope of modulation. The bandwidth of the second signal varied. The figure shows variation in the distance with respect to the bandwidth of signal $\theta_{2}$.
  The results are shown in Figure 4. We observe that the distance varied rather slightly around 1.4142 ($\sqrt{2}$) (less than 0.1% variation). This is a situation corresponding to Equation (44).
- Scenario 5: Analysis of the spectral distance function $f_{\theta_{1},\theta_{2}}\left(\nu \right)$ for two signals with different bandwidths.
  The parameters are described in Table 5. The signals had different bandwidths, but they had same phase, same middle frequency and same slope of modulation. We drew two figures: one representing the two signal spectra and the other representing the spectral distance between the two signals.
  The value of the Fisher–Rao distance between the two signals was $d_{L2,B}\left(\theta_{1},\theta_{2}\right)≃1.5455$. Figure 5 and Figure 6 show the impact of the signals’ frequencies on the distance. We observe that the spectral distance varied significantly within the common bandwidth.
- Scenario 6: Analysis of the spectral distance $f_{\theta_{1},\theta_{2}}\left(\nu \right)$ for two signals with different middle frequencies.
  The parameters are described in Table 6. The signals had different middle frequencies, but they had the same phase, same bandwidth and same slope of modulation. We drew two figures: one representing the signal spectra and the other representing the spectral distance between the two signals.
  The value of the Fisher–Rao distance between the two signals was $d_{L2,B}\left(\theta_{1},\theta_{2}\right)≃1.4142$. Figure 7 and Figure 8 show the impact of the signals’ frequencies on the distance. We observe that the spectral distance varied significantly within the common bandwidth. This is a situation corresponding to Equation (44).
- Scenario 7: A toy problem to demonstrate the usefulness of using the Fisher–Rao distance for parameter estimation.
  We had three signals. They had the same middle frequencies, same bandwidth and same slope of modulation, but they had different phases. The problem consisted of estimating the optimal phase shift values for the three signals to facilitate their discrimination during observation.
  In order to optimally separate the signals, we wanted to ensure the maximum possible value of the Fisher–Rao distance between any two signals. To achieve this, we calculated all the Fisher–Rao distances between the signals, namely $d_{L2,B}\left(\theta_{1},\theta_{2}\right)$, $d_{L2,B}\left(\theta_{1},\theta_{3}\right)$ and $d_{L2,B}\left(\theta_{2},\theta_{3}\right)$, and we determine the minimum distance:
  (49) $$d_{min}=\min\left(d_{L2,B}\left(\theta_{1},\theta_{2}\right),d_{L2,B}\left(\theta_{1},\theta_{3}\right),d_{L2,B}\left(\theta_{2},\theta_{3}\right)\right)$$
  For the signal $\theta_{1}$, the phase shift $\eta (\theta_{1})$ was arbitrarily set to zero. We looked for the respective values of $\eta (\theta_{2})$ and $\eta (\theta_{3})$, which determined the maximum value of $d_{min}$.
  The parameters of the scenario are described in Table 7.
  Figure 9 shows the variations in $d_{min}$ as a function of both parameters $\eta (\theta_{2})$ and $\eta (\theta_{3})$.
  The maximum value of the minimum Fisher–Rao distance $d_{min}$ was equal to $\sqrt{3}≃1.7321$, and it was reached for $\left(\eta \left(\theta_{2}\right),\eta \left(\theta_{3}\right)\right)=\left(2\pi /3,4\pi /3\right)$, or symmetrically, $\left(\eta \left(\theta_{2}\right),\eta \left(\theta_{3}\right)\right)=\left(4\pi /3,2\pi /3\right)$.
  When we attempted to fix the maximum value for the distance between two signals, such as $d_{L2,B}\left(\theta_{1},\theta_{2}\right)=2$, then another distance became less than $\sqrt{3}$.
  The optimal separation of the three signals was achieved when the phase difference between them was 2π/3, which is a well-known result in communication theory.

## 8. Conclusions

In this article, we addressed signal processing through information geometry by computing the Fisher–Rao distance between two finite-energy signals observed in Gaussian noise. To obtain a closed-form expression for this distance, one must express and then solve the geodesic equation, given the boundary conditions imposed by the signals under consideration. To formulate the geodesic equation, the Fisher information matrix was used and differentiated with respect to the parameters to obtain the Christoffel symbols.

The calculation starts from the multivariate normal distribution, and we re-derive the geodesic equations for both distributions with unknown means and covariance matrices, as well as for the case of a known covariance matrix. The geodesic equation can be solved in the latter case, allowing application to finite-energy signals. Through the frequency-domain model of these signals, we identified the statistical manifold L2(B) and obtained the closed-form expression for the Fisher–Rao distance on this manifold. This value appeared as a minimum bound for the Fisher–Rao distance between two finite-energy signals, regardless of the parameterization of these signals that defined a sub-manifold. The obtained expression makes it possible to highlight the spectral distance function $f_{\theta_{1},\theta_{2}}\left(\nu \right)$, which characterizes the impact of each frequency component on the Fisher–Rao distance value. We show that the Fisher–Rao distance varied as a function of the square roots of the signal-to-noise ratios, and for normalized signal-to-noise ratios equal to one, the distance value lied between 0 and 2. It is also shown that in the case of signals with disjoint spectral bands (or whose phase differences varied significantly within the band), the value of the distance was close to $\sqrt{2}$.

Several numerical examples related to LFM impulse signals are wenalyzed to illustrate the results obtained. It was shown that it is possible to reach the limiting values of the distance; the value zero was achieved for identical signals (confirming the concept of distance), and the value two was achieved for identical signals in opposition of the phase. Beyond these extreme cases, the distance varied more or less significantly around the value $\sqrt{2}$, depending on the phase characteristics of the signals and the separation of their frequency bands. Using a toy problem, we provide theoretical insights into signal estimation, with an open perspective toward clustering and machine learning applications, where geometric distances can characterize classification and estimation tasks.

In the numerical analysis, signals with a strictly equal duration *T* and without delay were considered. The influence of delays and the signal duration could be the subject of future research. Nevertheless, we anticipate that the Fisher–Rao distance between finite energy signals will help the characterization of signal databases.

## Figures and Tables

**Table 1:** Values of the parameters for scenario 1.

Parameters	*η*	*f*_0_	*ϵ*	*B_FM_*
***θ*_1_**	0	0.25	+1	0.25
***θ*_2_**	0	0.25	+1	0. ⋯ 0.5 ^1^

^1^ Variation between the values.

**Table 2:** Values of the parameters for scenario 2.

Parameters	*η*	*f*_0_	*ϵ*	*B_FM_*
***θ*_1_**	0	0.25	+1	0.25
***θ*_2_**	0	0.25	−1	0. ⋯ 0.5 ^1^

^1^ Variation between the values.

**Table 3:** Values of the parameters for scenario 3.

Parameters	*η*	*f*_0_	*ϵ*	*B_FM_*
***θ*_1_**	0	0.25	+1	0.25
***θ*_2_**	*π*	0.25	+1	0. ⋯ 0.5 ^1^

^1^ Variation between the values.

**Table 4:** Values of the parameters for scenario 4.

Parameters	*η*	*f*_0_	*ϵ*	*B_FM_*
***θ*_1_**	0	0.15	+1	0.25
***θ*_2_**	0	0.25	+1	0. ⋯ 0.5 ^1^

^1^ Variation between the values.

**Table 5:** Values of the parameters for scenario 5.

Parameters	*η*	*f*_0_	*ϵ*	*B_FM_*
***θ*_1_**	0	0.25	+1	0.25
***θ*_2_**	0	0.25	+1	0.15

**Table 6:** Values of the parameters for scenario 6.

Parameters	*η*	*f*_0_	*ϵ*	*B_FM_*
***θ*_1_**	0	0.25	+1	0.25
***θ*_2_**	0	0.15	+1	0.25

**Table 7:** Values of the parameters for scenario 7.

Parameters	*η*	*f*_0_	*ϵ*	*B_FM_*
***θ*_1_**	0	0.25	+1	0.05
***θ*_2_**	0 ⋯ 2*π* ^1^	0.25	+1	0.05
***θ*_3_**	0 ⋯ 2*π* ^1^	0.25	+1	0.05

^1^ Variation between the values.

## Data Availability

The original contributions presented in this study are included in the article. Further inquiries can be directed to the corresponding author.

## Abbreviations

The following abbreviations are used in this manuscript:

DFT	Discrete Fourier transform
FM	Frequency modulation
LFM	Linear frequency modulation
